# Elysian Happenings*Readers of The Red Back will readily recognize the characters in this masterly satire upon modern medical ethics, and they can give it local application, apply the cap to those it fits. Substitute “Austin, Texas.” “Red Back,” certain District Councillors, etc., wherever the reader thinks they come in. We all know the “Chesty Director” and “Dr. Knifem,” and some of you may know “Dr. Fightem” and recognize the asbestos paper.—D.

**Published:** 1907-05

**Authors:** 


### Elysian Happenings.*

### I.

The scene is laid in the Elysian fields where Hippocrates, the Father of Medicine, Harvey, the discoverer of the blood-circulation, who, while on earth, was considered and called a quack, Paracelsus, the brilliant and erratic physician of the sixteenth century, are having a quiet little game and incidentally discuss medical matters of today. They decide to visit the earth and look around. The focus of their special interest is Knockville, a town far-famed on account of its distinguished medical men and institutions. Under the leadership of Dr. Fightem, editor of the "Knockville Scrapper," an unconventional medical publication, they attend a meeting of the Knockville Mutual Admiration Society, where they get an ocular demonstration of the sham-ethics practiced by the so-called "big guns" in the profession. They witness a typical farce-comedy of professional life and get a chance to study some of the types of medical gentlemen, towit: Dr. Flatus, an exotic egotist who married money and thinks he has brains because he has money; Dr. Knifem, whose claim to immortality consists in being the greatest involuntary humorist in the Knockville profession, and known to fame as "the man with the aseptic peak"; Dr. Homunculus, a medical merchant who trusts nobody and incidentally is trusted by nobody, and many other types of medical men. The Elysian visitors are thoroughly disgusted. Harvey and Paracelsus have returned to the abode of the blessed, while Hippocrates is still in Knockville, enjoying the hospitality of the undismayed Dr. Fightem.

Dr. Fightem was busily engaged in looking over his correspondence and incidentally preparing some manuscript for the next

---

*Readers of The Red Back will readily recognize the characters in this masterly satire upon modern medical ethics, and they can give it local application, apply the cap to those it fits. Substitute "Austin, Texas," "Red Back," certain District Councillors, etc., wherever the reader thinks they come in. We all know the "Chesty Director" and "Dr. Knifem," and some of you may know "Dr. Fightem" and recognize the asbestos paper.—D.

issue of the "Knockville Scrapper." Hippocrates was standing by, watching the editor at work. Said he:

"Dr. Fightem, you carry your enthusiasm for the classic days of Hellas to a really pathetic degree. I see that you even refrain from using modern paper in preparing your literary products for the modern press. You prefer to use the papyrus of old, thick and indestructible. How do you manage to get your supply of papyrus?"

"My dear Father of Medicine," replied Dr. Fightem. "This is not papyrus, but a distinctly modern product, known as asbestos. I have recently begun to use asbestos for my manuscripts for the 'Knockville Scapper.' I owe the suggestion to my friend, Dr. Apis Duplex, Professor of Mental Diseases at the college. He is full of good ideas. He advised me to use some fire-proof material because the editorial matter for the 'Scrapper' was rather hot stuff."

"It could not possibly be too hot, my dear Fightem. Conditions in Knockville are distinctly septic and undergoing very active decomposition. They tell me that heat is considered a good germ-killer nowadays. Give it to them hot, the hotter the better. Pure gold will withstand the fire of purgation. As long as I am to pose as the Father of Medicine, I certainly do not wish to have anything but pure gold in my family. What I have seen here in Knockville leads me to think that there is much spurious metal in my profession. Not all is gold that glisters. In my time the practitioners of the healing art were required to pledge themselves by an oath which was named after me. Your 'big ones' interpret the oath of Hippocrates in a manner that is calculated to make Hippocrates swear."

While Hippocrates spoke there was a knock on the door. Dr. Fightem bade the knocking individual enter. A square-shouldered, able-bodied individual, with an affable smile and an aggressive hand-shake, entered. Dr. Eightem introduced the newcomer to his antiquated guest.

"This is my friend Dr. Rhachitis, one of the unterrified. Rhachitis, shake hands with Hippocrates, ex-health officer of antiquity."

"I am glad to meet you, Dr. Hippocrates. Your name is familiar. Didn't I meet you at the annual banquet of the Yaihoo County Medical Society three years ago? Guess I am mistaken. Let's see! Yes, yes, yes! I have come across your name in my bibliographic researches. Hang it! My memory is not as good as it used to be. Did you not make the first operation for stricture of the lower portion of the left ureter? You closed the meatus in the bladder and made an anastomosis of the ureter and the descending colon. This brilliant operation enabled the laboratory man to examine the excretion of the two kidneys separately, the urine from the left kidney being discharged per rectum. It was, indeed, a glorious achievement. Think of the tremendous diagnostic importance of rectal micturition! I have performed the operation seventy-six times myself. Incidentally I beg to say that I have given you full credit in my last volume on 'The surgery of the lower end of the left ureter.' The work is not complete as yet. It will be in five volumes. After I complete it, I expect to devote my time to the exhaustive study and investigation of the pathology, surgery and bibliography of the middle third of the left ureter. I shall continue these important historical and scientific researches until I have completed my cyclopedic work on the entire left ureter. This I expect to hand down to posterity as the precious heritage of a busy life. I have no doubt that you are still interested in the art and science of medicine and surgery. I operated on a man yesterday who had 671½ gall stones in his gall-bladder. While I was at it, I made a prophylactic appendectomy, removed his redundant prepuce with my own circular circumcision-knife which I take pleasure in showing you, restored the esthetic appearance of the anal orifice by clipping off a few unsightly external hemorrhoids which might have caused trouble later on, took a little lipoma off the man's back to prevent the possibility of future malignancy, gave the patient a world of relief by removing two ingrown toe-nails and hardly had time to get rid of a few sebaceous cysts of the man's head with elegance and dispatch when my assistant informed me that the chloroform had given out. I tell you we are busy people when we get started. I shall be pleased to have you spend the day with me and witness the irresistible progress of surgery."

Dr. Rhachitis stopped for a moment to catch his breath. This gave Hippocrates a chance to say:

"Dr. Rhachitis, I thank you for rescuing my name from hopeless obscurity and for giving me credit for some of my modest efforts. Men like you are rare. When I was a teacher of medical lore in Athens I had a pupil named Ascanides who was a most enthusiastic searcher for scientific truth. He was industrious, modest, and devoted his time to clearing up abstruse scientific problems. He left two large volumes on the 'Linea alba.' In this work he showed with admirable preciseness and irrepressible logic why the pudendal hirsute covering the man has a tendency to invade the linea alba. This is a most important biologic problem which has a distinct bearing on the social-economic aspect of sex. It was men like Ascanides who laid the foundation upon which the stately edifice of medicine was erected. I have no doubt that all the honors that are due a pathfinder in science have come to you."

"My dear Hippocrates," interjected Dr. Fightem, "your experience during the meeting of the Mutual Admiration Society ought to have taught you how much appreciation and recognition a man can expect in Knockville. It is an aggravated case of the prophet in his own country. In Knockville everybody is supposed to build his own monument. Dr. Flatus, for instance, is aware of this fact. For this reason he has tried to hypnotize the people and profession of Knockville into believing that a new hospital should be built for the poor, whose hardships he has taken very much to heart. These hardships are the stepping-stones to the great crowning effort of his life: a medical marble palace in Affenthal, our swell suburb, as a citadel in which Dr. Flatus shall reign supreme, as a monument to the greatest concentration and incarnation of superlative nihility in modern times. Fortunately this monument will not be built. No one can fool all the people all the time. There was an explosion in Knockville the other day. They say 170 pounds of dynamite let go. I do not believe it. They should investigate more closely. Who knows but what Dr. Flatus in looking for a site for his marble palace and monument became so inflated that he, i. e., the flatus, exploded. Dr. Rhachitis should not look for laurels in Knockville. Ambition, industry and achievement make a man an object of suspicion in Knockville."

Dr. Rhachitis replied modestly:

"Dr. Fightem is very kind, and I thank him. He certainly knows. But, gentlemen, I came near forgetting the purpose of my call. I have here a very interesting specimen which I exhibited before the members of the Mutual Admiration Society the other night. I showed it and asked them to make a diagnosis. Dr. Homunculus, who can hear the grass grow and can tell the difference between a male and a female pediculus without looking, took my specimen in his hand, looked at it, felt it, smelled it, tasted it, and pronounced it a rare variety of a vermiform appendix of man. I told him that he was wrong. He offered to bet me $1000. I accepted the bet, and then I informed him that the specimen was the caudal appendix of a rodent, commonly known as a rat-tail. I produced the rat to show whence the specimen had been taken. Dr. Homunculus, who is not a short-skate but a dead game sport, at once paid me the money. I immediately announced that I would spend the $1000, by paying for a dinner to be given at the best hotel in Knockville in honor of the fiftieth anniversary of the Mutual Admiration Society. I invited all the members, and have came to extend the invitation to Dr. Fightem and his illustrious guest. Dr. Homunculus has expressed a desire to have Dr. Fightem sit next to him at the dinner. Fightem, Homunculus just loves you. I believe he could eat you up. Take care so that he does not make a mistake at that dinner."

Dr. Fightem replied:

"The love of Dr. Homunculus is the great joy of my life. I just like to press his hand and look into those true, honest eyes that are the mirrors of a child-like, undefiled soul. Hippocrates and myself will certainly attend that dinner."

With a bow and friendly wave of the hand, Dr. Rhachitis left. After he had gone, Dr. Fightem said:

"There is a meeting of the three directors of the Knockville Hospital in progress at present. How would you like to attend it? It may not furnish inspiration, but certainly some amusement."

"Very well," said Hippocrates. "Wear this amulet so that we may not be visible to the gaze of mortal man."

He raised his hand. After a few moments Hippocrates and Dr. Fightem found themselves in the meeting room of the three directors of the Knockville Hospital.

"Why, bless my soul," whispered Hippocrates. "There is the fellow with the aseptic peak, halo, hammer, intellectual countenance, and all. He has grown some stouter since I saw him last."

"Don't think he has. Whenever he appears in all his glory as a director of the hospital, he swells up and looks chesty.* You notice that we have three directors. There were three graces in antiquity. Some say that *dis*-grace has visited the good old town of Knockville in a triple form. This is an uncharitable sentiment, however, which I do not share. See the little fellow with the Roman nose who is standing on a soap box so that he can be seen? He is the marvel of the age, the world-renowned Dr. Ike Burke. Be sure and notice the classic cast of countenance, and do not fail to be impressed with the Ambrosial locks. He trains them in imitation of Thundering Jove, whose Olympic curls Homer thinks so much of. Dr. Ike Burke has his impressive locks trimmed once

*We all know him.

in a while, i. e., when he can get one of his admiring friends to put up a quarter for this purpose. His name and the Roman nose are a trifle at variance. He is variously known as the 'man with the gum shoes,' or 'Ikey the meddlesome midget.' The word 'midget' does not refer to his intellect, which is mighty. He takes a large view of everything, especially of himself. When he was appointed medical director of the Knockville Hospital he refused to accept the congratulations of his friends. He insisted that the town of Knockville is the proper recipient of congratulations at being able to secure the services of so eminent and scholarly a man. He is a man of tremendous executive ability. He introduced gum shoes to be worn by the meddlesome directors. The latter, like the ghost of Hamlet's father, seem

"Doomed for a certain term to walk the night."

They meander noiselessly through the musty corridors of the hospital. Dr. Ike Burke is an expert at this. The sight of the great small man strikes terror into the hearts of the internes. When they do not see him they laught at him. But, you know, they are too young to appreciate true greatness. Listen! He is started to speak."

Dr. Ike Burke noiselessly moved about on the soap box, thanks to his gum shoes. There was explosive energy in his voice when he said:

"During my last nocturnal excursion through the corridors of the hospital I distinctly noticed the smell of cigarettes. I have informed the internes that whoever was caught with a cigarette in his *ponim* would be fined one dollar each time. The *mezumme* thus collected would be used to buy gum shoes for the directors."

With astounding agility Ikey jumped off the soap box and sat down in his chair.

"Dr. Ike Burke," opined Dr. Fightem, "is not a bad fellow at heart; in fact, he is more to be pitied than to be scorned. He is trying to fill a place that nature never intended for him. He is a bad actor, because he has a string tied to him. The fellow who holds the other end of the string pulls and tugs persistently. The result is many an ungraceful act and attitude on the part of the little fellow with the aquiline nasal proboscis. Dr. Ike Burke's actions as director of the hospital are not actuated by his interest in the hospital, by his humanitarian attitude towards the poor of the city, by his scientific enthusiasm, or by his sense of responsibility as a public official. He watches the string and the fellow that holds it. His body and soul belong to the string-holder.

In this respect he is in the same boat with Dr. Knifem, who is also a puppet controlled by a string. These two strings represent the rope by which the medical interests of Knockville will be choked to death, Ike Burke and Knifem serving as the involuntary executioners. Destroy these two strings and put the puppets out of the way. Thereon depends the medical future of Knockville. The third one in this famous trio, Dr. Kelly, popularly known as the Pride of Killarney, is helpless, even if he had the good-will to do what is right. He is a good fellow, but in awfully bad company. '*Mitgefangen, mitgehangen,*' as his friend with the aseptic peak would say. Dr. Kelly has arisen to speak. Let us listen!"

Dr. Kelly looked at his two associates earnestly, and began:

"God bless you! I have two matters to bring up before you. In view of the great number of unfortunates who are brought to our hospital suffering from the effects of alcohol, I have directed the superintendent to hang up a picture of our dear St. Patrick over each bed. This will help to keep out or drive out the snakes. So much for the scientific part of my work. The other matter concerns the integrity of this board and the honor of its individual members. Dr. Fightem in the 'Knockville Scrapper' has publicly stated that this board is controlled by 'bosses,' and that we are miserable figure-heads. The worst of it is, that this view is shared by nearly the whole medical profession of Knockville. I, for one, am not willing to be considered a tool or a puppet. I want the people and profession of Knockville to know that we are serving them and their interests, and that no 'boss' can control us. To prove this let us establish a merit-system and appoint the members of the staff accordingly. Let the whole profession be represented on this staff, not merely one clique or gang. Let us get rid of men who are incompetent or lazy. Let scientific work be done as it should be in a town of the size of Knockville. Let us no longer be the laughing stock of the people. Let us show that this board is not without honor, conscience, intelligence, manhood!!!"

A ghastly pallor was seen to spread over Knifem's contenance. He jerked convulsively. The string to which he was tied became tense. Somebody was pulling at it forcibly.

*"Was ist los,"* solicitously inquired Dr. Ike Burke, fondling and stroking Knifem's aseptic peak, which was wet with cold perspiration.

"I move that we adjourn," gasped Dr. Knifem. "I have an important interview with Dr. Homunculus, who has just now sent me a message by string, I mean wire. *Ach du lieber Gott!* What a headache!"

He made a sudden move for the door. A dog at the sound of his master's voice could not move any faster. Dr. Ike Burke opined smilingly:

"Business is Geschäft. Knifem knows his business, or they would not have put him on this board. I know *my* business. Kelly, you better come with us. There may be something in it for you. It is two against one, anyhow."

Kelly told him that he was sick and tired of the whole situation. Conscience makes cowards of all, sometimes even of a hospital director.

"Hippocrates, what do you think of this *status rerum?* Do you think that Dr. Kelly will yield and betray his trust?" queried Dr. Fightem.

"If he has any backbone, he will refuse to have anything to do with boneless acrobats," replied Hippocrates. "I have often wondered how it feels to look like a man without being one. I suppose Dr. Knifem could inform me. He knows everything."

"Dr. Knifem could give you information on a variety of subjects in connection with our hospital. The tribe of the Knifems is well and favorably interwoven with its unwritten history. I hope to have an opportunity soon of handling an exhaustive historical research down to posterity on this subject. Today's meeting of this board seems to be over. Let us be gone."

Hippocrates saw the fleeing forms of the two string-tied nonentities disappear in the distance: He said:

"Fightem, I am beginning to understand you. I am sure if these two nothings were not nothings they would have never been put where they are. To be nothings is what makes them useful to those whose miserable puppets they are. They could not fool any one with common sense. The whole situation is really laughable. How does one of your modern poets express it?

" 'Such labored nothings, in so strange a style,
Amaze the ignorant, while those that know just smile.' "

—*From International Journal of Therapy, Cincinnati, O.*

